# Postoperative Acute Submandibular Sialadenitis: A Case Report

**DOI:** 10.31729/jnma.8238

**Published:** 2023-08-31

**Authors:** Mona Sharma, Sandeep Bohara, Stuti Manandhar, Lumu Manandhar, Shyam Krishna Maharjan

**Affiliations:** 1 Department of Anesthesia and Intensive Care, Kathmandu Medical College and Teaching Hospital, Sinamangal, Kathmandu, Nepal; 2 Department of Neurosurgery, Kathmandu Medical College and Teaching Hospital, Sinamangal, Kathmandu, Nepal; 3 Kathmandu Medical College and Teaching Hospital, Sinamangal, Kathmandu, Nepal

**Keywords:** *airway obstruction*, *case reports*, *sialadenitis*

## Abstract

Acute postoperative sialadenitis is a rare complication usually after surgery involving extreme head and neck rotation, such as posterior fossa surgery. It is characterized by the development of swelling in the submandibular region, usually contralateral to the surgical side, either immediately or within hours post-operatively. We report a case of a 43-year-old woman who developed sialadenitis leading to upper airway obstruction in the postoperative period. Further, she developed bilateral neck and face swelling. Dexmedetomidine used as an infusion throughout the surgery could be an additional cause. Swelling without signs of inflammation is rapidly progressive and may cause airway obstruction. Therefore, awareness and recognition are important, as a delay in airway securement can cause a complete collapse of the airway.

## INTRODUCTION

Acute submandibular sialadenitis is a rare complication following posterior fossa surgery, the incidence of which has been assumed to be <1-2%.^1^ It is characterized by rapid facial and neck swelling, due to displacement of airway structures and airway edema which can lead to rapidly progressive airway obstruction and even potential death.^2^ Here we present a case of a 43-year-old woman with acute submandibular sialadenitis following left retromastoid suboccipital craniotomy leading to progressive airway obstruction and subsequent intubation.

## CASE REPORT

A 43-year-old woman presented with a history of headaches, nausea, and loss of balance while walking. Contrast-enhanced magnetic resonance imaging was suggestive of left vestibular schwannoma. The patient was planned for left retromastoid suboccipital craniotomy and excision of the tumour in a sitting position.

The patient was induced with fentanyl 2 mcg/kg, propofol graded and vecuronium 0.1 mg/kg. The patient was intubated with a 7.5 mm cuffed endotracheal tube. Cormack Lehane (CL) grade was IIa. Anaesthesia was maintained with dexmedetomidine infusion, propofol infusion and isoflurane at minimum alveolar concentration (MAC) 0.6 to achieve bispectral index (BIS) of 40. The patient's head was fixed in the Mayfield clamp and the patient was slowly put in a sitting position, no hemodynamic disturbance was noted. The neck was flexed; the face was slightly rotated to the right. Two-finger width between the chin and thorax was maintained and rechecked after final positioning. Under neuronavigation, suboccipital craniotomy was done, and gross total excision of the tumour was achieved, the tumour was firm arising from a left eighth nerve in the internal acoustic meatus extending up to the left cerebellopontine angle and compressing the brain stem. The tumour was attached to cranial nerve VII and was resected under the guidance of motor-evoked potential monitoring of VII nerve.

The surgery lasted 7 hours, following which the patient was extubated after confirming she was fully awake and obeying commands. After 30 min, the patient developed mild stridor. ,A small firm mass was noticed in the right submandibular region. The mass was non-tender, and hard and the skin appeared normal over the swelling. The diagnosis of submandibular gland swelling was made after assessment by bedside ultrasound. There was no paresis of the vocal cord on examination with ultrasound. Swelling in the submandibular area was increasing and the patient developed upper airway obstruction therefore, the patient was re-intubated with CL grade IIIa, and bougie-guided intubation was done with a 7 mm cuffed tube. The patient was kept in mechanical ventilation. After 6 hours patient developed massive swelling of the entire face more on the right. The swelling was progressive for 2 days. On day 2, the entire face and neck were swollen and tense with skin erosion on the neck. Bedside ultrasound revealed that the submandibular was enlarged and muscle and soft tissue appeared oedematous. The right internal jugular vein and external jugular vein size were considerably reduced. But there was no thrombosis and patency was maintained.

A computed tomography scan of the neck was performed on the 5^th^ day which showed an enlarged and oedematous right submandibular gland and surrounding tissue. The patient was started on steroids, and antibiotics from day one. After the third day, the swelling started to decrease. The patient did not have any leak around the endotracheal tube with cuff deflation. A cuff leak test was performed every day. Eventually, on the 6^th^ postoperative day patient had a positive leak test and the facial swelling had decreased significantly and hence was extubated. The patient was discharged on the 16^th^ day. Her recovery was uneventful henceforth.

## DISCUSSION

The exact cause of postoperative sialadenitis is elusive. One thing that is common in these theories is extreme positioning of the head and neck and it develops in the immediate postoperative period. Most of the patients require immediate intubation to secure the airway. There can be severe oedema of epiglottis and arytenoid cartilage and shift of the airway making intubation impossible and hence emergency tracheostomy could be required.^2^ Prolonged neck rotation and flexion in these surgeries can lead to bilateral obstruction of the jugular vein, which obtunds venous return from head and neck leading to further neck and face swelling. Despite obstruction to the flow of jugular veins, brain swelling did not occur in most cases, as compensatory venous drainage exists via venous anastomoses between sigmoid and vertebral plexus i.e. mastoid and emissary veins. And those patients without well-developed anastomoses could develop brain oedema.^3^ As the neck is in a neutral position and the body in the supine position after the surgery; gravity-induced blood flow and elevation in blood pressure induce and increase the swelling. Moreover, extreme rotation leading to pressure ischemia for a prolonged time or compression of the facial artery and reperfusion of ischemic tissue could lead to oedema.^4^ There have been reports of mediastinal swelling, vocal cord palsy, Horner's syndrome and brachial neuropathy.^5^ A bilateral submandibular swelling, which resulted in the death of the patient, has also been reported.^6^

The swelling of the submandibular gland swelling can occur as ductal obstruction can lead to stasis and oedema. The submandibular gland duct can also be compressed by an endotracheal tube, fixed on the same side as the Wharton's duct open on the sublingual papilla at the base of the tongue.^7^ Sympathetic nervous system activation due to any reason can lead to increased viscosity and secretion, with no outlet leading to stasis. In our case, there was stimulation of the VII cranial nerve around the medulla pons border may have further increased the secretion of the salivary gland, whereas the flow may have been decreased due to dexmedetomidine infusion, an alpha 2 agonist, which may have contributed to the swelling as it has antisialagouge properties.

In our case, the patient had bilateral neck and facial swelling, which differs from other cases where unilateral swelling has been reported. Dexmedetomidine can be an added culprit to extreme head positioning.

The swelling usually reduces by the third or fourth day and overall good prognosis, but few have reported deficits.^8^ Adequate hydration, sialagogues, and steroids have been used to reduce inflammation and empirical antibiotics are usually preferred as stasis can invite infection by Gram-positive cocci.^9,10^ None of these treatments are proven to decrease swelling.

Awareness among anaesthesiologists and intensivists is the key to reducing morbidity. As early airway management saves a life. Measures to reduce its incidence, starting from refraining from extreme head rotation and minimal use of antisialogogues cannot be overemphasised.
